# Interpreting the kinematic theory of rapid human movement as an optimal control theory

**DOI:** 10.3389/fnhum.2026.1685216

**Published:** 2026-05-28

**Authors:** Najoua Assila, Ben Braithwaite, Mickaël Begon, Réjean Plamondon

**Affiliations:** 1 Chair of Psychology of Learning and Instruction, Faculty of Psychology, School of Science, Technische Universität Dresden, Dresden, Germany; 2 Centre for Tactile Internet with Human-in-the-Loop (CeTI), Technische Universität Dresden, Dresden, Germany; 3 Département de Génie électrique, Polytechnique Montréal, Montréal, QC, Canada; 4 School of Kinesiology and Exercise Sciences, Faculty of Medicine, University of Montréal, Montréal, QC, Canada; 5 Centre de recherche Azrieli du CHU Sainte-Justine, Montréal, QC, Canada

**Keywords:** kinematic theory of rapid human movement, motor control, movement invariants, optimal control theory, velocity profile

## Abstract

Human rapid movements often exhibit stereotypical patterns and invariants, which are essential for understanding movement control. The Kinematic Theory posits that invariances observed in velocity profiles are inherent to the structure of the neuromuscular system. In contrast, optimal control theory assumes that invariances result from optimality. Although the lognormal velocity profiles proposed by the Kinematic Theory produce the most realistic rapid movements, they have yet to be examined from an optimality standpoint. Our objective was to investigate the implication of velocity profile lognormality through the lens of optimal control theory. To this end, we analyzed commonly used kinematic control strategies using the lognormal parameters of the Kinematic Theory to identify the key factors contributing to rapid movement cost: energy efficiency, movement smoothness, and time. We then constructed an original composite cost function and applied it to predict the control strategy of an arm model (1 or 2 degrees of freedom) during a rapid extension. Our composite cost function successfully produced asymmetric velocity profiles that resemble a lognormal profile more closely than other control strategies, although the beginning of the movement remains too rapid. We further examined our composite function using experimental data of one participant performing 29 fast arm extensions. We showed that the optimal end-effector velocity profile matched the experimental measurements as closely as a lognormal profile. By analyzing the optimal predictions of the velocity profile, we drew links between the components of the composite function and the lognormal parameters. Finally, we proposed strategies to further improve the optimal control predictions.

## 1 Introduction

Human rapid aimed arm movements exhibit highly invariant characteristics. These invariances include a velocity profile with a dominant asymmetric peak and a trajectory that is nearly straight (Morasso, 1981; Plamondon, 1995a). Several theoretical models have been proposed to explain how these invariances emerge.

The Kinematic Theory of rapid human movements attributes these invariances to the intrinsic structure and organization of the neuromuscular system (NMS) (Plamondon, 1995a,b). From the motor cortex to the muscles, the neural command is transmitted through a series of subsystems organized both hierarchically and in parallel (Ghez et al., 1991). To account for this structure, the cumulative time delay after each subsystem is assumed to be proportionally related to that of its predecessor (Plamondon et al., 2013a). As a result, the response of the NMS to the neural command, specifically the velocity of the end-effector, converges toward a lognormal function. This lognormal model forms the basis of the Kinematic Theory (Plamondon et al., 2003). More advanced models, such as the delta-lognormal or the sigma-lognormal, incorporate the coordinated activity between agonistic and antagonistic sub-systems (Plamondon and Guerfali, 1998) and replicate more evolved behaviors (O'Reilly and Plamondon, 2009; Martín-Albo et al., 2015).

The Kinematic Theory provides a global analytic representation of the kinematics of a well-learned rapid movement. Unlike other models, such as the equilibrium point hypothesis (Feldman, 1986; Feldman and Levin, 2009), it does not rely on the mechanical properties of the NMS, nor on the architecture of the neural network. Instead, it emphasizes the temporal organization of the NMS. This allows the Kinematic Theory to circumvent intricacies associated with the NMS dynamic modeling while still preserving predictive power. This modeling choice is supported by multiple studies suggesting that the NMS primarily controls movement through kinematic variables rather than kinetic ones (Wolpert et al., 1995; Plamondon et al., 2003; Djioua and Plamondon, 2010). Velocity profiles predicted by the Kinematic Theory replicate key experimental results on fast movements, particularly the asymmetric shape of the main velocity peak (Plamondon, 1995a; Plamondon et al., 2003; Lebel et al., 2017). The Kinematic Theory predictions have been substantiated through a number of empirical studies involving over 11,000 participants. These studies encompass a range of effectors (e.g., eye, head, trunk, wrist, fingers) and have been conducted under diverse experimental conditions. Taken together, these studies span more than 300,000 velocity patterns, with sampling frequencies ranging from 15 to 240 Hz (Plamondon, 2020).

Optimal control theory proposes an alternative interpretation of invariants in human rapid movement. It posits that the NMS operates in the most efficient way possible, *i.e*., its movement is associated with both a cost and a set of constraints (Umberger and Miller, 2017). Consequently, the system responds to stimuli by selecting solutions that minimize its execution cost. Applied to biomechanical systems, optimal control theory follows a two-step process. First, a mechanical model is defined to represent the NMS properties, along with the interactions among its subsystems. This mechanical model is governed by dynamic and kinematic equations, with defined control and state variables. Next, a cost function is introduced to express the control strategy and assess the performance of candidate solutions. Defining a cost function that produces human-like movement remains a major challenge, yet it is essential for understanding the behavior of the NMS (Mombaur et al., 2010; Berret et al., 2011).

Different control strategies have been proposed to explain human movement. For rapid movements, feedforward control is dominant, making these movements incompatible with optimal feedback control approaches (Todorov and Jordan, 2002). Harris and Wolpert proposed that the control strategy minimizes the variance of the end-effector's final position (Harris and Wolpert, 1998)—a principle shown to be equivalent to minimizing effort under the assumption of signal-dependent noise in the motor command (Diedrichsen et al., 2010). Other commonly used control strategies employ either purely kinematic variables (such as velocity, acceleration, or jerk), kinetic variables (such as torque or torque derivative), or a combination of both (Berret et al., 2011). In robotics or when interested in joint load during human movements, kinetic models can be more relevant thanks to their direct link to actuators (Khoukhi and Hamam, 1992; Jazar, 2010). However, to study the kinematic characteristics of the movement, kinematic control strategies are generally more practical and relevant (Mainprice et al., 2016).

As the Kinematic Theory approaches the NMS as an end-effector velocity generator, it provides limited insights into multiple-joint control and the resolution of kinematic redundancy—issues that can be directly explored through the optimal control approach. Nevertheless, integrating these two frameworks could provide the advantage of incorporating the NMS organization (*i.e*., the lognormality of velocity) in the formulation of optimal control strategies. Such a synthesis can improve our understanding of joint redundancy control within the broader hierarchy of the NMS. Additionally, it would facilitate the examination of the interaction between the NMS organization and the transition from feedback control to feedforward adaptation during childhood (Malone et al., 2025), as well as the links between temporal and biomechanical representations of movement. Despite the evident benefits of bridging the gap between the Kinematic Theory and optimal control theory, combining these theories is particularly challenging owing to their fundamentally different approaches to modeling the generation of rapid human movement. Interpreted through the lens of the Kinematic Theory, the velocity profiles generated by typical kinematic-based optimization models can be understood as simplified versions of the lognormal model (Djioua and Plamondon, 2010). However, the Kinematic Theory has yet to be examined through the framework of optimal control theory, leaving a gap in unifying these approaches. Given that the Kinematic Theory provides one of the most robust analytical models of rapid human movements, we aim to investigate the lognormality from an optimality standpoint. First, we interpret commonly used kinematic control strategies—such as the minimum jerk (Flash and Hogan, 1985), the minimum time (Engelbrecht, 2001), and the geodesic (minimum energy) models (Neilson et al., 2015)—in relation to the lognormal function described in the Kinematic Theory. Then, by investigating how the lognormal parameters influence these kinematic cost functions, we propose a new composite cost function. This function is used to predict the kinematics of a rapid arm extension. The resulting velocity profiles are first compared to the lognormal profile, then to experimental data. Through this comparison, our overarching goal is to further draw links between the Kinematic Theory and optimal control strategies.

## 2 Methods

### 2.1 Optimal control theory: common control strategies

#### 2.1.1 Minimum squared derivative models

The NMS and human movement are generally described by their kinematic characteristics (Wolpert et al., 1995). Therefore, creating optimal control strategies based on kinematics is relevant. A popular type of cost function relies on the squares of the time derivatives of the movement:


$$\begin{array}{l} C\left(k,\;x\right)=\int_{t_{start}}^{t_{end}}{\left(\frac{d^{k}x}{dt^{k}}\right)}^{2}dt\; \end{array}$$ (1)

where *x* is the position variable, *k* is the derivative order, *t*_*start*_ and *t*_*end*_ are the movement's start and end. Depending on the order of the derivative, this kind of cost optimizes different characteristics of the movement:

- *k* = 1 minimizes movement velocity. Combined with inertial parameters, it minimizes kinetic energy (Hatze and Buys, 1977).
- *k* = 2 minimizes movement acceleration. This cost is associated with optimizing energy transfers, *i.e.*, the forces exerted during the execution of the movement (Nelson, 1983). This model is unable to produce a plausible acceleration profile (Berret et al., 2011).
- *k* = 3 minimizes movement jerk to find the smoothest motion possible (Uno et al., 1989). It smooths muscle torque changes, putting less stress on the joints (Klein Breteler et al., 2002).
- *k*≥4 minimizes higher-order derivatives. As the physical interpretation is less evident (Eager et al., 2016), these higher order derivatives are rarely used in biomechanics.

Among these models, the minimum jerk (or its kinetic equivalent) produces the most realistic movements (Klein Breteler et al., 2002), albeit not reproducing all the invariant characteristics of rapid human movement, *e.g.*, the asymmetry of the velocity profile (Djioua and Plamondon, 2010).

Composite cost functions were also explored to produce more realistic movements. Using linear combinations of minimum squared derivatives-type functions, Berret et al. (2011) implemented an inverse optimal control approach to identify the major contributors to the cost of human movement. Their findings indicated a combination of energy and jerk as the best cost function for pointing gestures. Ben-Itzhak and Karniel (2008) proposed a model combining jerk and acceleration, which demonstrated superior performance in predicting realistic human movement compared to only minimizing jerk. The potential of composite models represents a promising avenue that we further explore in this study.

#### 2.1.2 Minimum time

Minimum squared derivative models do not usually consider the complex structure of the NMS (Djioua and Plamondon, 2010), although some studies have tried to account for it (Engelbrecht, 2001). When assessing rapid human movements, execution time is a relevant factor in the movement cost. Participants are usually instructed to perform the movement as quickly as possible, and experimenters may also impose strict time constraints (Meyer et al., 1988). Therefore, the conscious cost that the participant associates with rapid human movements partly comprises movement time. The minimum-time model supposes a bang-bang control, *i.e.*, with instantaneous transitions between maximum acceleration and deceleration phases. Since the resulting velocity profile is unrealistic, it has been suggested to use the bang-bang signal as input to the NMS. This signal is processed by the NMS, which is modeled by a series of low-pass filters representing synapses (Baldissera et al., 1998). Mathematically, the NMS can be modeled by the convolution integral of these filters' responses, with the velocity profile determined by the NMS's impulse response (Engelbrecht, 2001). After *n* filter, the response velocity profile exhibits an asymmetric Gamma profile (Djioua and Plamondon, 2010). Nevertheless, as the low-pass filters are considered independent, the velocity profile converges to a Gaussian as the number of filters increases (Djioua and Plamondon, 2010). The minimum-time model suggests that considering response time could produce asymmetric velocity profiles.

### 2.2 Movement derivatives and movement time in a Kinematic Theory paradigm

The lognormal model of the Kinematic Theory predicts a causal velocity profile characterized by its asymmetric bell shape. This profile depends on the amplitude of the motor command D and the time of occurrence of the motor command *t*_0_, the logtime delay μ, and the log response time σ (see Supplementary material for the mathematical definition). In particular, the logtime delay μ quantifies the cumulative time delay of the system on a logarithmic scale, capturing the temporal delay between the central command and the peak end-effector response. The log response σ represents the response time of the system on a logarithmic scale, and it captures the dispersion of the NMS transmission delays. Together, these lognormal parameters (μ and σ) provide insights into the velocity profile's characteristics (Supplementary material). Their values vary slightly depending on the type of movement (Lebel et al., 2017). But overall, σ controls the asymmetry of the system, whereas μ governs the underlying activation dynamics. For further details on these parameters, readers are referred to (Plamondon 1995b), Plamondon et al. (2003).

As the temporal organization of the velocity profile is fully controlled by the combination of μ and σ, examining how these lognormal parameters affect kinematic-based optimization costs is sufficient to investigate the Kinematic Theory from an optimality perspective. Accordingly, we will evaluate the norm of the velocity and jerk cost functions (*k*∈[1, 3] in Equation 1) and the movement time using the lognormal model. We will limit our analyses to μ∈[−3.0, 1.5] and σ∈[0.1, 0.6], values that are compatible with arm movements (Djioua and Plamondon, 2009; Woch et al., 2011; Pan et al., 2019).

#### 2.2.1 Effect of the lognormal parameters on the velocity profile

**Figure 1:**
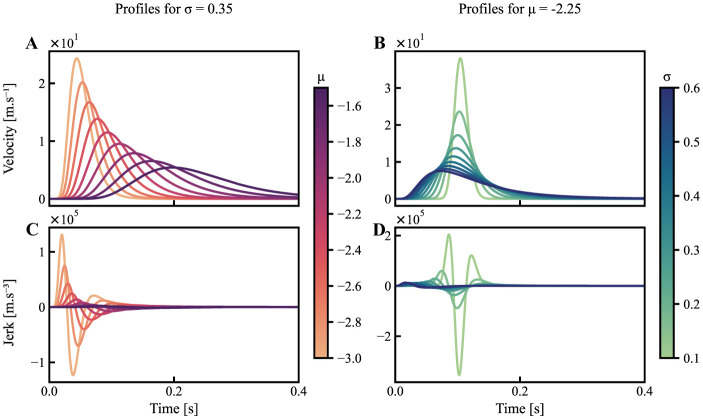
Effects of lognormal parameters (left: μ and right: σ) on the velocity **(A,B)** and jerk **(C,D)** profiles.

As μ decreases (*i.e.*, its magnitude increases as it is negative), the velocity mode is reached faster, and its peak magnitude increases (Figure 1A). Since exp(μ) represents the median, the time required to cover half of the planned distance, and it fixes the limit of the cumulative time delays. Consequently, a higher peak velocity results in shorter movement execution time. In contrast, when σ increases, the movement takes longer to complete (Figure 1B): the velocity profile widens, the peak velocity decreases, and the average velocity increases. Assuming a constant logtime delay μ, these changes in the velocity profile suggest that the responses of the NMS sub-systems are of smaller magnitude but last longer. This implies a shift toward more parallel functioning sub-systems, which aligns with the observed increase in skewness as σ increases, indicating that slower movements tend to be more asymmetrical.

#### 2.2.2 Effect of the lognormal parameters on the jerk profile

**Figure 2:**
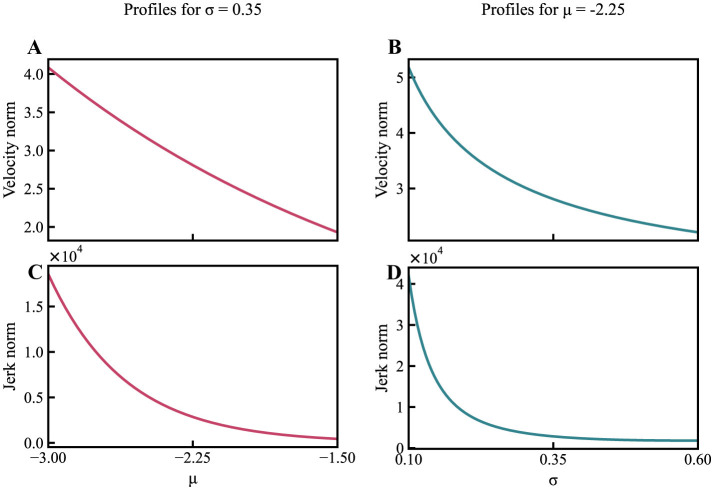
The 2-norm of the velocity **(A,B)** and jerk **(C,D)** profiles, presented in Figure 1, as a function of the lognormal parameters (left: μ and right: σ).

As μ decreases, the peaks of the jerk profile increase and occur earlier (Figure 1C). Increasing σ reduces the jerk amplitude and extends the movement duration (Figure 1D). The jerk-related cost decreases when μ or σ increases, with a greater impact of σ (Figures 2C, D). Fast movements require reaching a high maximum velocity in a short time, *i.e*., sudden applications of large forces to start and end the movement. Hence, when the time needed to perform half of the movement [*i.e*., the median exp(μ)] decreases, the cost associated with the jerk increases. Similarly, a smaller σ implies faster NMS sub-systems, *i.e.*, they produce their maximum force fast, producing more jerk.

The monotonic decrease in velocity and jerk costs with increasing μ and σ suggests that, in the absence of additional constraints or cost functions, such models would inherently favor slow movements. Therefore, to generate realistic movements, it becomes essential to incorporate additional cost functions that actively penalize high values of μ and σ.

#### 2.2.3 Movement time

The last cost considered here is movement duration. In a purely lognormal velocity profile, the end-effector velocity never reaches zero, requiring a practical definition of when the velocity is sufficiently small to be considered zero (Plamondon et al., 2007; Djioua and Plamondon, 2009; Martín-Albo et al., 2015). On a logarithmic timescale, 99.7% of the movement occurs within the interval (μ−3σ, μ+3σ). Translating this to a linear timescale, 99.7% of the movement takes place between exp(μ−3σ) and exp(μ+3σ). Accordingly, the movement time can be defined as follows:


$$\begin{array}{l} MT=t_{end,\;99.7}-t_{start,\;99.7}=2*\exp\left(\mu \right)*\sinh\left(3\sigma \right) \end{array}$$ (2)

This definition offers the advantage of linking the time characteristics of the velocity profile to the lognormal parameters through a simple mathematical relationship, without requiring planning in advance the movement duration, which is controlled by μ and σ. Notably, movement time is independent of *t*_0_ (*i.e.*, the time occurrence of the motor command). Instead, *t*_0_ simply shifts the velocity profile temporally, equally influencing the start ($t_{start,\;99.7}=t_{0}+e^{\mu -3\sigma }$) and end ($t_{end,\;99.7}=t_{0}+e^{\mu +3\sigma }$) of the movement.

While the objective is to identify a set of μ and σ that minimizes movement time, it is also desirable for the movement to begin as early as possible after the motor command. Interestingly, increasing σ prolongs movement time, but leads to an earlier movement onset (*i.e.*, smaller *t*_*start*, 99.7_), whereas increasing μ delays the start. As the σ also influences the skewness of the velocity profile, its optimal value must balance movement duration with asymmetry of the velocity curve. Through Equation 2, we can deduce that both movement time and *t*_*end*, 99.7_ increase exponentially with μ and σ, which contrasts the evolution of minimum squared derivatives costs (Figure 2). This observation supports the idea that rapid human movements are likely governed by a compromise between minimizing kinematic costs and reducing movement time.

### 2.3 Human fast movement as a trade-off between movement time, energy, and smoothness

The abovementioned descriptions of the cost functions highlight the impossibility of using a single cost function to optimally control human movements. Non-trivial optimal behavior emerges when opposing forces are balanced, resulting in a minimum of the cost function within the system's feasibility space. Here, this space encompasses all possible end-effectors' movements generated by the NMS. Since the NMS attempts to produce the smoothest possible movements, jerk is considered a key factor in movement generation (Flash and Hogan, 1985). In addition, Berret et al. (2011) have shown that kinetic energy plays an important role, as it reflects the mechanical energy required for movement execution. Finally, as the goal of rapid movements is to minimize execution time, movement time should also be considered as a contributing factor (Engelbrecht, 2001). Supported by these considerations, we hypothesize that the NMS behavior results from a trade-off between smoothness, energy efficiency, and movement time. In the following, we introduce a cost function that captures this compromise and evaluate it within the Kinematic Theory framework. This new cost function is inspired by the expression used to evaluate the response time of the NMS sub-systems (Djioua and Plamondon, 2004):


$$\begin{array}{l} C_{comp}=t_{end,\;99.7}\cdot \sqrt{\int_{t_{start}}^{t_{end}}\left[\alpha^{*}v^{2}+\beta^{*}j^{2}\;\right]dt} \end{array}$$ (3)

with *v* the velocity of the movement and *j* its jerk. The relative weights α and β determine the contributions of kinetic energy and jerk to the composite function, while ensuring dimensional consistency. To balance the amplitudes of velocity and jerk, we set $\left(\frac{\alpha }{\beta },\beta \right)=\left(5000,\;1\right)$ (Supplementary material, Equations S1.2, S1.3). Since this study focuses solely on movement kinematics, we do not incorporate torques in the kinetic energy definition. Instead, we evaluate the system's kinetic energy using the square of the velocity. Additionally, we fix the bounds of the integral, as the velocity profile is, by definition, negligible outside (*t*_*start*, 99.7_, *t*_*end*, 99.7_). To examine the behavior of this new composite cost function within the Kinematic Theory paradigm, we evaluate it as a function of the lognormal parameters μ and σ. Furthermore, we compare it to two simplified models: a minimum energy and time criterion [*C*_*v*.*t*_ = *C*_*comp*_(β = 0)] and a minimum jerk and time criterion [*C*_*j*.*t*_ = *C*_*comp*_(α = 0)].

**Figure 3:**
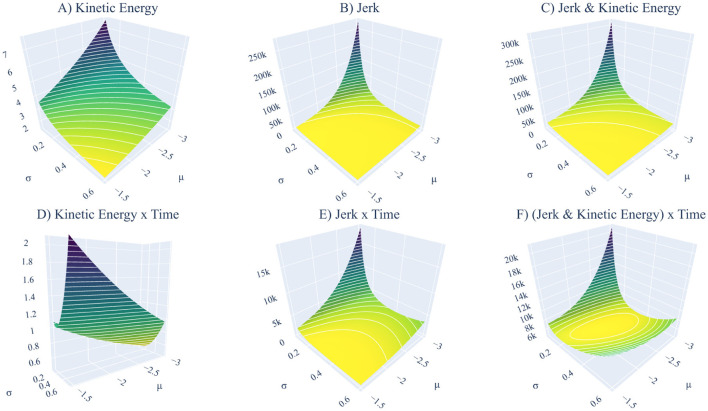
Behavior of the cost functions with respect to μ and σ: **(A)** Kinetic energy. **(B)** Jerk. **(C)** Composite cost using jerk and kinetic energy with α/β = 5, 000. From **(D–F)**, the cost functions were modified (“x Time”) to account for the movement end time (*t*_*end*, 99.7_), see Equation 3. The two horizontal axes represent μ and σ, while the vertical one and the color gradient represent the magnitude of the cost function: the darker the color, the larger the cost function. The white contour lines indicate cost function iso-values.

Incorporating movement time into the cost functions considerably alters their behavior (Figure 3). For instance, unlike the pure energy cost (Figures 2A, B), the energy–time cost increases monotonically with μ and σ (Figure 3D). The inclusion of time in the jerk cost function leads to the emergence of an optimal σ value for each given μ within the physiological range (Figures 3B, E). Most notably, for our composite cost function (Equation 3), the inclusion of time leads to the existence of an optimal set of μ and σ that minimizes the cost (Figure 3F). Interestingly, the location of this minimum, primarily with regard to μ, can be adjusted with the ratio α/β: higher ratio values shift the optimal μ to smaller values. Thus, our composite cost function makes it possible to find an optimal operating point within physiological bounds for μ and σ, which was impossible for the other kinematic cost function (Figures 3A–E).

### 2.4 Prediction of arm extension kinematics using the novel composite function

#### 2.4.1 The composite function as an optimal control objective function

As the premises of the Kinematic Theory and optimal control differ, this new cost function cannot be directly applied to solve an optimal control problem. Specifically, within the Kinematic Theory framework, the cost function inherently imposes constraints on the velocity and jerk profiles (*i.e*., the lognormal function). Accordingly, it is necessary to define a cost function that minimizes movement time, velocity, and jerk while favoring an asymmetric velocity profile. Based on *C*_*comp*_ and the definition of the response time of a system [*i.e*., the response spread on the time scale, which is equivalent to σ^2^ (Brown, 1963)], we reformulate *C*_*comp*_ to be used for an optimal control problem (OCP), as follows:


$$\begin{array}{l} C_{OCP}=\int_{t_{start}}^{t_{end}}\left[\frac{\alpha }{\beta }v^{2}+j^{2}\;\right]\cdot t^{2}dt \end{array}$$ (4)

This revised cost function should favor asymmetrical outcomes, as it places less weight on costs incurred at the start of the movement compared to those at the end. Finally, it is important to note that the Kinematic Theory describes the transfer function linking velocity to neural command. In contrast, an optimal control problem formulated using kinematic states and controls (Equation 4) does not explicitly account for the delay between the motor command and its kinematic manifestation. Accordingly, *C*_*OCP*_ should be adjusted to express this causality (Equation 5):


$$\begin{array}{l} C_{OCP}=\int_{t_{start}}^{t_{end}}\left[\frac{\alpha }{\beta }v^{2}+j^{2}\;\right]\cdot {\left(t-t_{0_{OCP}}\right)}^{2}dt \end{array}$$ (5)

where *t*_0_*OCP*__ represents a time shift parameter. This parameter will ensure congruence between the temporal spaces of the two premises, facilitating the comparison of their predicted velocity profiles.

#### 2.4.2 Mechanical arm model

**Figure 4:**
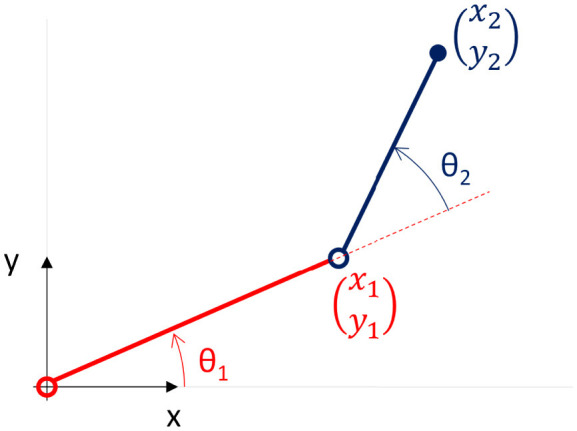
A Diagram for the 2-DoF rotating arm model. The elbow and wrist have the coordinates (*x*_1_*, y*_1_) and (*x*_2_*, y*_2_), respectively.

We will investigate two mechanical arm models. The first has a single degree of freedom (1-DoF) rotation in the horizontal plane. It can represent a forearm drawing a curved line or waving to someone. The second model is a 2-DoF upper limb actuated at the shoulder and elbow. The 1-DoF model is a sub-section of the 2-DoF model (Figure 4). The length of the arm segments are *L*_1_= 0.30 and *L*_2_= 0.28 m, with corresponding masses *m*_1_= 2.4 and *m*_2_= 1.8 kg, representing the upper arm and the forearm + hand, respectively (de Leva, 1996). To compute the segmental moments of inertia, each segment was modeled as a rigid cylinder with a radius of 3 cm and a uniform mass distribution.

#### 2.4.3 Investigated theoretical cases

We constrained the range of motion slightly below the physiological limits of the shoulder and elbow joints. Upper and lower bounds for velocity, acceleration, and jerk were defined according to experimental data of fast reaching movements (Pigeon et al., 2003). We simulated a rapid extension movement of the elbow and shoulder (*i.e.*, the arm starts in a folded position and ends up fully open). Three cases were investigated:

- **Case 1**: A 1-DoF (θ_1_) with constrained initial and final states. In this case, the angular state $\left(\theta_{1},\;\theta_{1}\right)$ and the end-effector kinematics (s,s) are interchangeable for evaluating the kinematic state cost function, as they are proportional $\left(s=\;L_{1}.\theta_{1}\;\right)$.
- **Case 2**: A 2-DoF (θ_1_, θ_2_) with constrained initial and final states. The velocity and jerk components of the cost function were computed in the joint space (θ_1_, θ_2_).
- **Case 3**: Similar to Case 2, but the cost function was evaluated in task space, using end-effector kinematics, which implicitly couples the proximal and distal joints.

#### 2.4.4 Optimal control problem and solver

To find the optimal solution, we used the cost function described previously (Equation 6):


$$\begin{array}{c} C_{OCP}\left(q,\;\alpha ,\;\beta \right)=\int_{t_{start}}^{t_{end}}\left[\frac{\alpha }{\beta }{*v\left(q,\;t\right)}^{2}+{j\left(q,t\right)}^{2}\;\right]*\\ {\;(t-t_{0_{O}CP})}^{2}dt \end{array}$$ (6)

where *q* is the state vector, and *t*_0_*OCP*__ = 0.01 *s*. We did not impose any kinetic constraints on the model to limit our analysis to the kinematic outputs. We also opted to use joint jerks as controls (*u*≡*j*) for improved dynamic consistency (Puchaud et al., 2023). The complete problem can be written as follows (Equation 7):


$$\begin{array}{l} \underset{q,\;u}{\min}C_{OCP}\left(q,\;\alpha ,\;\beta \right) \end{array}$$ (7a)

$$\begin{array}{l} \text{subject to}\\ \forall t,-110^{•}{\leq \theta }_{1}\left(t\right)\leq 30^{•},5^{•}{\leq \theta }_{2}\left(t\right)\leq 140^{•}\\ \forall t,|{\overset{.}{\theta }}_{1}\left(t\right)|\;<\;600^{•}/s,|{\overset{.}{\theta }}_{2}\left(t\right)|\;<600^{•}/s\\ \forall t,|{\overset{..}{\theta }}_{1}\left(t\right)|\;<\;5000^{•}/s^{2},|{\overset{..}{\theta }}_{2}\left(t\right)|\;<5000^{•}/s^{2}\\ \forall t,|{\overset{\mathrm{...}}{\theta }}_{1}\left(t\right)|\;<\;4000^{•}/s^{3},|{\overset{\mathrm{...}}{\theta }}_{2}\left(t\right)|\;<4000^{•}/s^{3} \end{array}$$ (7b)

The details of the problem constraints and boundary conditions can be found in the appendix (Table A1). Using a direct multiple shooting transcription, the optimal control problem was solved using Bioptim (Michaud et al., 2023) with 60 shooting points and a 4^th^-order *Runge-Kutta* integration with 5 intermediate steps. The non-linear program was solved using *Ipopt* (version 3.14.14), running with the MUMPS linear solver (Wächter and Biegler, 2006). The Hessian was calculated using the exact second derivatives, and the convergence tolerance was fixed at 10^−6^.

#### 2.4.5 Experimental data

In addition to the theoretical cases described above, we recorded 29 rapid arm-extension movements performed by a single participant with no history of neurological or musculoskeletal disorders. The volunteer provided written informed consent, and the experimental procedure was reviewed and approved by the ethics committee of the University of Montreal (CERC#2024-6063). Starting from a flexed shoulder and elbow position, the participant was instructed to extend their arm as quickly as possible in the horizontal plane. While an overall description of the final arm position was given, the participant was not constrained to a specific end state. Reflective markers on the shoulder, elbow, and wrist were used to track the arm motion (200 Hz). Movement onset and offset were defined as the times at which the wrist's linear velocity crossed 1% of its peak magnitude. The mean movement duration was 0.74 ± 0.05 s. The data were resampled over the average movement duration, and the mean initial and final states were used as boundary conditions for the optimal control problem. As in theoretical cases 2 and 3, we investigated the problem in both joint space (case 4) and task space (case 5).

#### 2.4.6 Analysis

First, we solved the three theoretical cases under different optimal criteria: minimizing solely jerk (*C*_*j*_); jerk and time (*C*_*j*•*t*_); ,or jerk, time and energy (*C*_*OCP*_). To relate the optimal control predictions to the Kinematic Theory, a lognormal function was fitted to each resulting velocity profile (Djioua and Plamondon, 2009). For this fitting, *t*_0_ was fixed to its value in the optimal control problem (*t*_0_*OCP*__), and σ was constrained within its physiological range. The similarity between each predicted velocity profile and its corresponding lognormal fit was quantified using the signal-to-noise ratio (SNR), where the lognormal profile served as the reference signal and the fitting residuals represented noise. Accordingly, a higher SNR indicated a better fit, and profiles with *SNR* > 15 were considered consistent with a lognormal (Plamondon et al., 2013b).

Using only the first case, we investigate the effect of the ratio α/β. This ratio modulates the location of the optimal movement with regard to the lognormal parameters μ and σ (Figure 3F). Since these parameters determine the movement end time of a lognormal profile (Equation 2), we anticipate that the interaction between the ratio α/β and *t*_*end*_ would influence the shape of the predicted velocity profile. Accordingly, we compared velocity profiles predicted by *C*_*OCP*_ for α/β = 5000 and α/β = 10^5^ when *t*_*end*_ was set to either 0.8 or 0.4 s. These values for the ratio α/β were set based on previous studies on arm movement (Ben-Itzhak and Karniel, 2008; Berret et al., 2011). For the 2-DoF cases, we have evaluated the state of the proximal and distal joints (*i.e*., joint angles and angular velocities), along with the effectors' behavior—specifically the Cartesian linear velocities of the elbow and wrist (Figure 4) and their trajectories in time.

Using the experimental data, we solved the optimal control problem for the last two cases (4 and 5) using the three optimality criteria. We fitted a lognormal function to the mean experimental wrist velocity profile. To compare the velocity profiles predicted by the lognormal model and the optimal control solutions with the experimental data, we examined their respective cumulative distribution functions and computed the Anderson–Darling distance (i.e., a lower value indicates a better match between the predicted and experimental cumulative distribution functions). This approach allowed us to account for the experimental variability, avoid favoring the lognormal model that was directly fitted to the data, and should be minimally affected by simplifications in the arm's mechanical model.

## 3 Results

### 3.1 Case 1: arm extension with a single DoF

**Figure 5:**
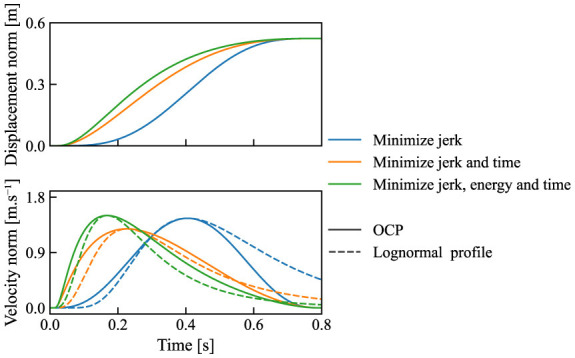
Displacement and velocity profiles predicted by different objective functions (solid line): minimum jerk (*C*_*j*_, blue); minimum jerk and time (*C*_*j*•*t*_, orange); and the composite function minimizing jerk, energy, and time (*C*_*OCP*_, green) with α/β = 5, 000. The lognormal velocity profiles fitted to each cost function prediction are represented in dashes.

**Figure 6:**
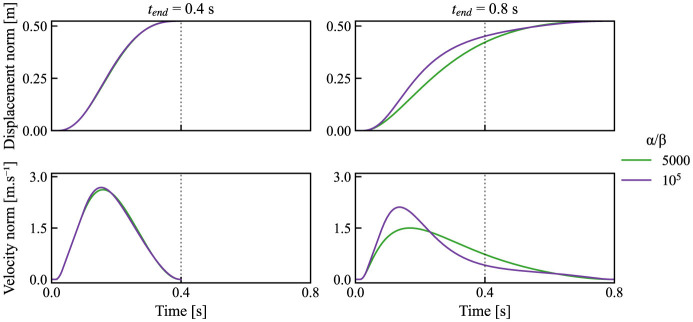
Effect of the ratio (α/β) and the movement duration (*t*_*end*_, columns) on the displacement **(top)** and velocity **(bottom)** profiles as predicted for the 1-DoF problem using the composite cost function (*C*_*OCP*_).

Each of the three cost functions used to solve the 1-DoF optimization problem produced distinct kinematic profiles (Figure 5). Minimizing jerk alone (*C*_*j*_) resulted in a symmetric velocity profile. In contrast, minimizing jerk and time (*C*_*j*•*t*_); or jerk, time, and energy (*C*_*OCP*_), produced asymmetric velocity profiles, *i.e.*, skewed toward the beginning of the movement. Moreover, the composite function (*C*_*OCP*_) led to faster acceleration and a more concave deceleration of the arm. The velocity profiles resulting from (*C*_*j*_), (*C*_*j*•*t*_) and (*C*_*OCP*_) were best fitted with lognormal profiles with parameters (μ, σ) of (−0.7, 0.45), (−1.12, 0.6), and (−1.42, 0.6), respectively. The associated SNRs were 10.51, 13.76, and 14.09 dB. The shape of the velocity by *C*_*OCP*_ was strongly influenced by both movement duration and the α/β ratio. Specifically, for shorter movement durations (*t*_*end*_), a higher α/β ratio was required to maintain a velocity profile similar to the lognormal function (Figure 6 and Table 1).

**Table 1:** Effect of the ratio (α/β) and the movement duration (*t*_*end*_) on the parameters (μ, σ) of the lognormal profile fitted to the velocity predictions for the 1-DoF problem using the composite cost function (*C*_*OCP*_), and their respective SNR.

α/β	t_end_(s)	μ	σ (0.1–0.6)	SNR (dB)
5,000	0.4	−1.48	0.6	11.90
5,000	0.8	−1.42	0.6	14.09
10^5^	0.4	−1.51	0.6	12.33
10^5^	0.8	−1.63	0.6	24.67

The logtime response bound was defined as (0.1, 0.6).

### 3.2 Case 2: arm extension with a 2-DoF model minimizing the cost function for the generalized coordinates

**Figure 7:**
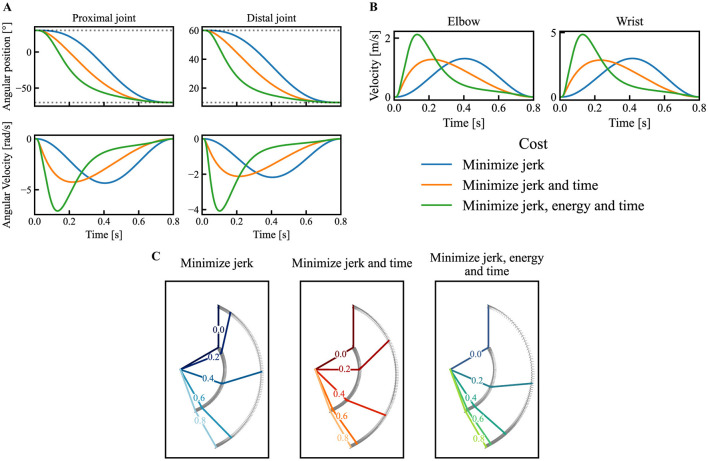
Simulation of a 2-DoF arm opening using the generalized coordinates' minimum jerk (*C*_*j*_, blue), minimum jerk and time (*C*_*j*•*t*_, orange) and composite function (*C*_*OCP*_, green) with α/β = 10^5^. **(A)** Angular position **(top)** and velocity **(bottom)** profiles for the proximal **(left)** and distal **(right)** joints. **(B)** Linear velocity norm profiles of the elbow joint **(left)** and end-effector (wrist, right). **(C)** Configurations of the arm at regular intervals (*t* = 0, 0.2, 0.4, 0.6, and 0.8 s) for the three cost functions. The increase in time is represented by an increase in the color saturation. The gray points indicate the effectors' trajectories.

When solving the optimal control problem for the 2-DoF model using generalized velocity and jerk as parameters of the cost functions, the angular velocity profiles of both joints were similar and synchronous, regardless of the cost function used (Figure 7). Qualitatively, the velocity profiles predicted from minimizing jerk alone (*C*_*j*_), jerk and time (*C*_*j*•*t*_) or the composite function (*C*_*OCP*_) closely resembled those predicted for a 1-DoF case, with asymmetry emerging when movement time was included in the cost (Figure 7B). Finally, the composite function (*C*_*OCP*_) predicted a faster arm extension, followed by a slower ending of the movement (Figure 7C).

### 3.3 Case 3: arm extension with a 2-DoF model minimizing the cost function for the end-effector

**Figure 8:**
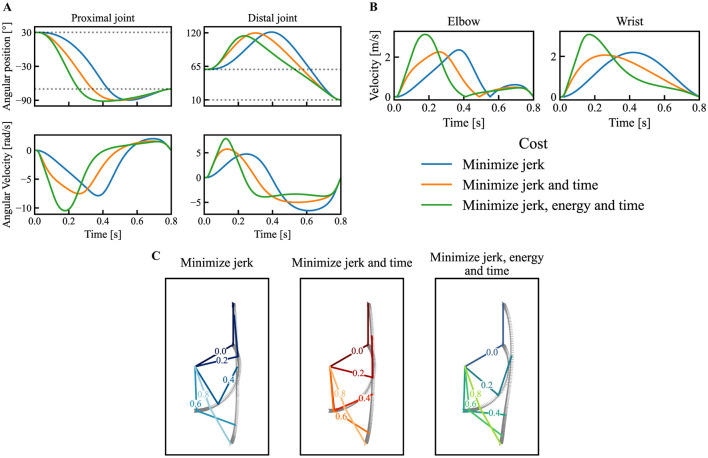
Simulation of a 2-DoF arm opening using the end-point's minimum jerk (*C*_*j*_, blue), minimum jerk and time (*C*_*j*•*t*_, orange) and composite function (*C*_*OCP*_, green) with α/β = 10^5^. **(A)** Angular position **(top)** and velocity **(bottom)** profiles for the proximal **(left)** and distal **(right)** joints. The horizontal dashed lines indicate the constrained states at the start and end of the movement. **(B)** Linear velocity norm profiles of the elbow joint **(left)** and end-effector (wrist, right). **(C)** Configurations of the arm at regular intervals (*t* = 0, 0.2, 0.4, 0.6, and 0.8 s) for the three cost functions. The increase in time is represented by an increase in color saturation. The gray points indicate the effectors' trajectories.

When evaluating the cost functions using end-effector kinematics, the predicted kinematics for the proximal and distal joints differed (Figure 8A). The proximal joint extended beyond the target angle before flexing during the second half of the movement. In contrast, the distal joint exhibited a nearly opposite pattern, initially flexing and transitioning to extension later in the task (Figure 8C).

In both cases, only the composite functions had *SNR*>15. However, compared to results derived using generalized coordinates, the overall velocity profile of case 3 deviated more from a lognormal function (Table 2), while its maximum linear velocity of the end-effector was reduced (Figure 7B vs. Figure 8B). Additionally, the linear velocity norm of the elbow crossed zero around 0.4 s, which was not observed in case 2. Finally, the end-effector's trajectory was less curved (Figure 8C).

**Table 2:** Parameters of the lognormal profiles fitted to the end-effector velocity profiles predicted for the 2-DoF problem using different cost functions calculated for the generalized coordinates (Case 2) or the end effector (Case 3), and the SNR values to quantify the similarity between the optimal control predictions and the lognormal profile.

	Cost function	μ	σ(0.1 − 0.6)	SNR (dB)
Case 2	Minimize jerk *C*_*j*_	−0.62	0.51	10.01
	Minimize jerk and time *C*_*j*.*t*_	−1.1	0.6	13.08
	Composite function *C*_*OCP*_	−1.69	0.6	22.43
Case 3	Minimize jerk *C*_*j*_	−0.51	0.6	10.56
	Minimize jerk and time *C*_*j*.*t*_	−0.99	0.6	13.50
	Composite function *C*_*OCP*_	−1.47	0.56	18.21

The logresponse time bound was defined as (0.1–0.6).

### 3.4 Case 4 and 5: experimental validation

**Figure 9:**
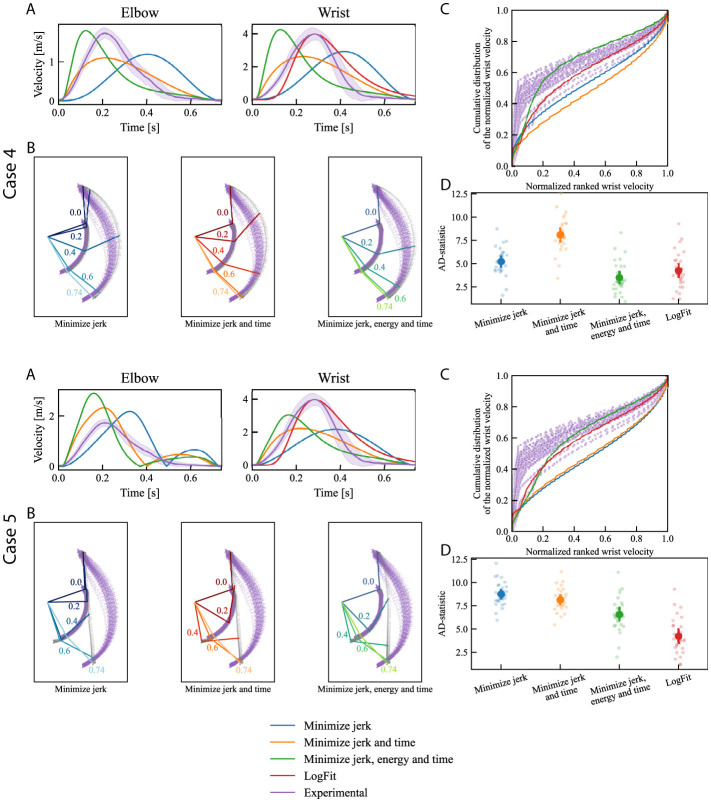
Predictions of the optimal control problem compared with experimental data (purple) in joint space (case 4, top) and task space (case 5, bottom). Three cost functions were considered: minimizing jerk (blue), minimizing jerk and time (orange), and our composite function (green; α/β = 10^5^). **(A)** Predicted linear velocity profiles of the elbow and wrist, with a lognormal profile fitted to the mean experimental data (LogFit, red). The experimental mean and standard deviation are shown. **(B)** Arm configurations at regular intervals (*t* = 0, 0.2, 0.4, 0.6, and 0.74 s) for the three cost functions; increasing color saturation denotes progression in time. Gray points indicate predicted effectors' trajectories, and purple points indicate experimental trajectories. **(C)** Cumulative distribution functions of the wrist linear velocity. **(D)** The Anderson–Darling statistic comparing predicted wrist velocity profiles with experimental ones. Light colored points represent the Anderson–Darling statistic computed for individual experimental trials, while the darker points and error bars denote its mean and 95% confidence interval.

The kinematic characteristics predicted by the optimal control solutions were comparable to those observed in cases 2 and 3. Specifically, the elbow's linear velocity norm crossed zero only in case 5, and the end-effector trajectories in case 5 (Figure 9A) were straighter than those in case 4 (Figure 9B). Examining the cumulative distribution function, the wrist linear velocities predicted using either the composite function (*C*_*OCP*_) or the lognormal model were the closest to the experimental data for both cases (Figure 9C). This was confirmed with the Anderson–Darling distance (Figure 9D), with the lognormal model (4.25 ± 1.94) and the composite function (case 4: 3.50 ± 1.71; case 5: 6.57 ± 1.85) having the lowest values. They were followed by the jerk-only criterion (case 4: 5.23 ± 1.47; case 5: 8.76 ± 1.30), and finally by the combined jerk-and-time criterion (case 4: 8.10 ± 1.87; case 5: 8.15 ± 1.23).

## 4 Discussion

### 4.1 Single and two DoF interpretation

In the 1-DoF problem, minimizing jerk produces an unrealistic symmetrical velocity profile, as expected (Viviani and Flash, 1995; Engelbrecht, 2001; Klein Breteler et al., 2002). In contrast, both the jerk-time (*C*_*j*•*t*_) and our composite (*C*_*OCP*_) cost functions produce an asymmetric velocity profile. As hypothesized, incorporating time in the cost function leads to movement asymmetry (*i.e.*, profile skewed toward the beginning of the movement). This aligns with the literature, as the minimum-time model produces asymmetric velocity profiles (Engelbrecht, 2001). Additionally, comparing the jerk-time cost function (*C*_*j*•*t*_) to the composite function (*C*_*OCP*_), the latter's velocity profile shows a more concave deceleration phase and a faster acceleration. This profile leads to a higher SNR for the composite function prediction as compared to that of the jerk-time prediction, as well as a logtimedelay closer to the physiological range. This suggests that the composite function may provide more physiologically plausible predictions. This is further supported by the improvement in SNR when the ratio α/β increases (Table 1), indicating that the energy contribution is fundamental to the control strategy.

While our composite function produces an inflection in the deceleration phase, the acceleration phase remains convex and unrealistically fast. Indeed, the weight introduced by the time renders the objective function almost null at the start of the movement, irrespective of predicted jerk or velocity values. Thus, the optimality criterion imposes minimal constraints at the beginning of the movement. Mechanically, this suggests that the current optimal control problem inadequately translates the difficulty of overcoming the inertia of an initially static arm. In contrast, the Kinematic Theory effectively captures rapid movement kinetics without explicitly accounting for the NMS inertial parameter, relying instead on the organization and structure of its subsystems (Plamondon, 1998). Previous research indicates that joint impedance varies during movement and may be controlled independently of muscle activation (Ludvig et al., 2012; Piovesan et al., 2013). Thus, a potential strategy to reduce the initial acceleration at movement onset would be to add joint impedance to the arm model as an additional control variable within the optimal control problem.

Irrespective of the movement time or the ratio α/β, the logresponse time (σ) of the fitted lognormal functions converges toward its upper bound of 0.6. This higher value results in a more skewed velocity profile and a longer response time. Additionally, the SNR of the optimal control velocity profile increases as the predicted μ decreases (Table 1), resulting in a higher maximal velocity and a smaller mode. This suggests that the feasibility space for the composite function (*C*_*OCP*_) may reflect a balance between the system's activation dynamics and its response time. Specifically, as the profile is more skewed, the system's response gets slower, so faster activation is needed to respect the task's time constraint (*i.e*., high acceleration observed at the start). This further highlights the importance of adding joint impedance control to our current kinematic model. These additional controls might account for muscle mechanics (*i.e.*, the increase of muscle force is associated with an increase in its stiffness), acting as low-pass filters, bringing the optimal control system closer to the rationale behind the kinematic theory.

The simulations (1-DoF and 2-DoF) show that our composite cost function produces more lifelike velocity profiles compared to those predicted with a minimum jerk model. This suggests that motor control involves a balance between motion smoothness and kinetic energy minimization. This finding aligns with the work of Berret et al. (2011), who, through an inverse optimal control investigation, found evidence for composite cost functions. They concluded that motor planning is dynamic, with task-dependent weightings of the different components of the cost function. This is coherent with our observation that shorter movement durations (*t*_*end*_) require higher α/β ratios to maintain an asymmetric velocity (Table 1 and Figure 6, bottom). Indeed, for a given movement duration, both the asymmetry of the velocity profile and the concavity of its deceleration phase increase with the ratio. This aligns with our initial analysis, where the jerk and time cost (*C*_*j*•*t*_) becomes increasingly dominant compared to the kinetic energy and time cost (*C*_*v*•*t*_) as movements become faster (*i.e*., μ and σ decreasing in Figures 3D, E). Therefore, a higher ratio is needed to keep the minima of the composite function (*C*_*comp*_) within a physiological range. It is important to note that within the Kinematic Theory, time is not an input parameter for modulating the shape of the velocity profile. Thus, the ratio α/β may need to be defined as a function of movement duration [*MT* = 2^*^exp(μ)^*^sinh(3σ)] to reflect the sensorimotor system's flexibility in adjusting planned motion to task-increased speed constraints. This suggests that the ratio α/β can serve as a tuning parameter to constrain the space of plausible solutions of the optimal control problem to the family of lognormal functions (*C*_*comp*_, Figure 3F).

### 4.2 2-DoF control: control coordinates- or task-oriented

When solving the 2-DoF problem using the generalized coordinates (case 2), the model predicts generalized velocities similar to those of the 1-DoF problem. Particularly, both proximal and distal joints have similar profiles (Figures 7A, B). Conversely, solving the 2-DoF problem using the end-effector velocity and jerk (case 3) leads to differences between the states of the two joints. Particularly, the elbow's linear velocity (Figure 8B) is similar to predictions that would be made with the delta-lognormal model—a generalization of the lognormal model accounting for the presence of both an agonist and antagonist sub-systems. Indeed, as we account for the inertial properties of the two segments, the model starts by flexing the distal joint, which brings the center of the mass of the arm closer to the proximal joint. Then, to reduce the jerk of the end effector, the model predicts different movement directions for the proximal (flexion) and distal (extension) joints. This results in a straighter trajectory of the end-effector compared to the one predicted for the first 2-DoF problem (case 2). These straighter paths are in line with the experimental trajectories drawn by participants pointing to different targets using 2-DoF (shoulder and elbow) (Morasso, 1981; Abend et al., 1982).

Solving the 2-DOF problem with a task-oriented control strategy (case 3) is more coherent with the Kinematic Theory, as it expresses that the control strategy treats both joints as sub-systems. However, the SNR of the end-effector velocity profiles for the task-oriented control (case 3) is not systematically higher than that of a joint-space control (case 2) for all cost functions. In particular, for the composite cost function, a task-oriented control leads to a slightly lower SNR. Hollerbach et al. (1987) noted that using staggered-time control in the joint space resulted in straighter trajectories of the end-effector, which is indicative of hand-space control. In our study, neither formulation accounts for the proportionality of time delays between joint responses, a key assumption of the Kinematic Theory, which might explain our SNR results (Plamondon et al., 2013a). Kaminski and Gentile (1986) suggested that a space-time transformation regulates the onset of joint movements. Accordingly, to account for the proportionality principle, the optimal control problem can be further constrained by linking *t*_0 < *uscore*>*ocp*_ of each joint to its displacement magnitude. Kaminski and Gentile (1986) further argued that such mapping leads to smoother velocity profiles. Thus, and as argued for the 1-DoF problem, incorporating joint impedance control may result in a sequential movement onset. Indeed, the proximal-distal sequence can be observed at different levels: from the neural command to the kinematics (Serrien and Baeyens, 2017). Additionally, its presence at one level contributes to the constraints that promote its emergence at the other levels. As the formulation of the optimal control problem does not dissociate these levels, the biomechanical origin of the proximal-distal sequence (*i.e*., transfer of momentum and energy) can be leveraged to simulate proportional time delays in response to the central command. The proximal-distal sequence can be interpreted as a manifestation of the self-organization of the NMS. This organization also underlies the lognormal nature of the velocity profiles, as described by the Kinematic Theory. Accordingly, accounting for this sequence in the formulation of the optimal control problem may inherently constrain its feasible solution space to the one predicted by the Kinematic Theory. Our analysis further highlights the generality of the Kinematic Theory. Indeed, drawing on the hierarchical organization of the NMS, it appears to implicitly account for the influence of both kinetic and kinematic constraints in the emergence of lognormality.

Examining experimental data, our composite function predicts velocity profiles that are as close to the experimental measurements as the lognormal model of the Kinematic Theory. The cumulative distribution functions are particularly similar when the composite function is minimized in task space (case 5) rather than joint space (case 4), supporting the importance of accounting for the proximal-distal coordination. Examining the end-effector trajectories further shows that the curvature of the experimental data lies between the predictions of case 4 (overly circular) and case 5 (overly straight). Interpreted in terms of joint impedance, this suggests that impedance modulation during task execution yields solutions that are neither purely joint-space nor purely task-space controlled, pointing instead to a role for biomechanical musculoskeletal constraints in restricting the solution space explored by the neural controller.

### 4.3 Further interpretation using the lognormal parameters

As the composite cost function was based on the lognormal function analysis, we can reinterpret the cost function in terms of the lognormal parameters of the Kinematic Theory. For a given movement of a given duration predicted by our composite cost function (*C*_*OCP*_), it should be possible to determine a set of possible values for (μ, σ)_*opt*_ as long as the ratio α/β enables the cost function to define a compromise between the movement kinetic energy and its jerk (Figures 3F, 6). A detailed analysis of the (μ, σ)_*opt*_ as a function of the movement duration and the ratio α/β can be found in the Supplementary material. The improved fit of the composite function response to the lognormal profile as a function of the ratio α/β for a given movement duration (Figure 6, Table 1, and Supplementary material) suggests the existence of a specific control strategy that optimally balances jerk and energy costs. Vu et al. (2016) highlighted that this balance may be associated with leveraging interaction torque to achieve a compromise between kinetic and kinematic efficiency. The utilization of interaction torque expresses the existence of a representation of limb dynamics employed during motor command generation (Gribble and Ostry, 1999). This representation may explain the proximal-to-distal temporal organization of joints observed in skilled movement (Serrien and Baeyens, 2017), a fundamental aspect of the Kinematic Theory. Thus, through this ratio, and as the velocity profiles grow closer to a lognormal impulse response, the composite control strategy may be capturing the internal model of limb dynamics, enabling the prediction of lifelike movement using solely kinematic controls.

Using our composite function, rapid movement control may be viewed as a two-level process. First, an appropriate ratio α/β is selected for a given movement, encapsulating the optimality of motor preparation while integrating limb dynamics, consistent with the thalamo-cortical circuit model proposed by Kao et al. (2021), which treats the primary motor cortex and the limb as a coupled system during motor preparation. Second, the composite cost with the selected ratio α/β is minimized, reflecting predictive processing to minimize effort and maximize movement smoothness within the given time constraints. Physiologically, the appropriate ratio α/β might express muscle short-range stiffness that ensures musculoskeletal stability during fast movement (de Vlugt et al., 2011), minimizing the need for feedback control.

As this study investigated a simplified 2D arm extension movement, additional research is needed to extrapolate our findings for more complex 3D movements, and to further corroborate the composite function against empirical data, using a more realistic representation of the degrees of freedom of the upper limb, as well as a larger sample size. Evaluating muscle and joint stiffness as a function of movement time is also needed to support our interpretation of the ratio α/β.

This study establishes links between optimal control theory and the Kinematic Theory by examining the properties of the lognormal model. Our analysis suggests that the optimality of the motor control is closely linked to the temporal organization of the NMS. This established relationship is crucial for understanding the interaction between the NMS temporal organization and the biomechanical reality of the musculoskeletal system. Such insights will promote a comprehensive approach to motor control, integrating perspectives from both neuroscience and biomechanics. We found that the Kinematic Theory can be combined with optimal control strategies to formulate a composite cost function that produces more realistic velocity profiles for simple, rapid human movements compared to other kinematic control strategies. The links between the two theories offer new opportunities for interpreting lognormal parameters, extending the Kinematic Theory to tasks dependent on feedback, and evaluating optimal solutions and control strategies. They also provide the groundwork for leveraging the principles of the Kinematic Theory to enhance current optimality-based control strategies.

## Data Availability

The raw data supporting the conclusions of this article will be made available by the authors, without undue reservation.

## Appendix

**Table A1:** Constraints and boundary conditions of the optimal control problem for the three considered cases.

	Case 1	Case 2	Case 3
States, controls	$q(\theta_{1},\theta_{1}),u\equiv \;{\dddot{\theta }}_{1}$	$q(\theta_{1},\theta_{2},\;\theta_{1},\theta_{2}),u\equiv ({\dddot{\theta }}_{1},\;{\dddot{\theta }}_{2})$	
Initial state	*t*_*start*_ = 0 *s* θ_1_(*t*_*start*_) = 30 $\theta_{1}(t_{start})0/s$	*t*_*start*_ = 0 *s* (_θ_1_, θ_2_)*t*_*start*__ = (30, 60) ${\left(\theta_{1},\theta_{2}\right)}_{t_{start}}(0/s,\;0/s)$	
End state	*t*_*end*_ = 0.8 *s* θ_1_(*t*_*end*_) = −70 $\theta_{1}(t_{end})\;0/s$	*t*_*end*_ = 0.8 *s* (_θ_1_, θ_2_)*t*_*end*__ = (−70, 10) ${\left(\theta_{1},\theta_{2}\right)}_{t_{end}}(0/s,\;0/s)$	
Velocity component **v**(**q, ** **t**)	$\theta_{1}(t)$	$\left(\theta_{1}(t),\;\theta_{2}(t)\right)$	$\frac{d}{dt}\left(f_{FwdKin}\left(q,\;u\right)\right)$
Jerk component **j**(**q, ** **t**)	${\overset{\mathrm{...}}{\theta }}_{1}\left(t\right)$	$\left({\overset{\mathrm{...}}{\theta }}_{1}\left(t\right),\;{\overset{\mathrm{...}}{\theta }}_{2}\left(t\right)\right)$	$\frac{d^{3}}{dt^{3}}\left(f_{FwdKin}\left(q,\;u\right)\right)$

**f**_**FwdKin**_ corresponds to the forward kinematics step necessary to compute the end-point's velocity and jerk from the problem's states and controls.
