# Supplementary material for: Interpreting the kinematic theory of rapid human movement as an optimal control theory
Source: Front Hum Neurosci. 2026 May 28;20:1685216. doi: 10.3389/fnhum.2026.1685216 (PMC13255677; doi:10.3389/fnhum.2026.1685216)
Supplement: Supplementary file 1 [file Supplementary_file_1.pdf]

1. The lognormal velocity profile

The lognormal velocity profile of the Kinematic Theory is defined as follows:

$$v(t)=D\Lambda(t, t_0, \mu, \sigma^2) \tag{S1.1}$$

where

$$\Lambda(t; t_0, \mu, \sigma^2)=\begin{cases} \dfrac{1}{\sigma\sqrt{2\pi}(t\text{-}t_0)} exp\left(\text{-}\dfrac{(ln(t\text{-}t_0)\text{-}\mu)^2}{2\sigma^2}\right) & if\ t_0<t \\ 0 & otherwise \end{cases} \tag{S1.2}$$

with D the amplitude of the motor command, t_0 the time occurrence of the motor command, μ the logtime delay, and σ the logresponse time.

The lognormal parameters (μ and σ) modulate the shape of the velocity profile. Key characteristics of this profile can be defined as follows:

- The median of the lognormal response, i.e., the moment when half the displacement due to the motor command was performed, is $t_0 + exp(\mu)$;
- The mode of the velocity profile, i.e., the time at which the velocity reaches its maximum value, is $t_0 + exp(\mu - \sigma^2)$;
- The time delay which provides a global evaluation of the rapidity of the NMS when reacting to the motor command is defined by $t_0 + exp\left(\mu + \frac{\sigma^2}{2}\right)$;
- The skewness, a measure of asymmetry, is $(exp(\sigma^2) + 2)\sqrt{exp(\sigma^2) - 1}$.

The jerk profile can be defined as the second derivative of Eq. S1.1:

$$j(t) = D\frac{w(t)^2 + 3\sigma * w(t) + 2\sigma^2 - 1}{\sigma^2 * (t - t_0)^2}\Lambda(t; t_0, \mu, \sigma^2) \tag{S1.3}$$

with

$$w(t) = \frac{\ln(t - t_0) - \mu}{\sigma} \tag{S1.4}$$

2. Conceptual interpretation of the composite function using the lognormal parameters

Using the 1-DoF problem for simplicity, we evaluated the lognormal parameters by fitting the lognormal curve (Supplementary material, Eq. S1.1) to the predictions of the composite cost function (C_{OCP}) for various movement durations (from 0.3 to 0.9 s), and for four values of the α/β ratios ([10^3, 10^4, 10^5, and 10^6], Fig S1). These ratios were set in reference to the ratio 10^5 predicted from experimental data of arm movement (Berret et al., 2011). By setting these values, we want to qualitatively explore the effect of favoring jerk or energy minimization in the control strategy. The dependency of the optimal set $(\mu, \sigma)_{opt}$ on the movement time and the ratio α/β is coherent with the dependency of these lognormal parameters on movement conditions change (Pan et al., 2019) and participants' age (Woch et al., 2011).

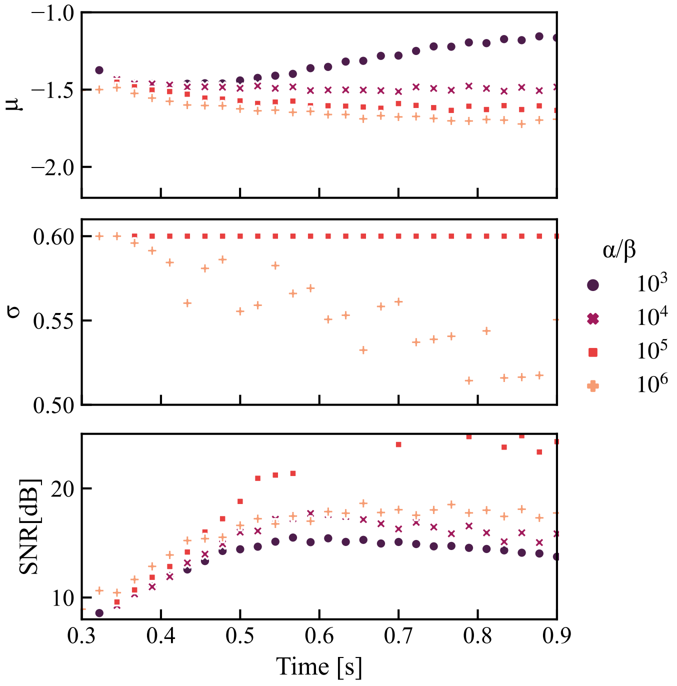

Fig S1. Optimal lognormal parameters based on the velocity profiles predicted by the composite cost function (C_{OCP}) for different α/β ratios using the 1-DoF problem, and their respective SNR.

Interestingly, there seems to be an optimal ratio α/β for which the fit between the composite function predictions and the lognormal function is highest. Particularly, for the four evaluated values, α/β=10^5 provides a composite cost function that predicts velocities closest to the Kinematic Theory. Good fits are obtained for movement durations around 0.6 s and get worse as movement duration decreases. This observation is consistent with our previous discussion on the prediction of the 1-DOF problem. Similar to the 1-DOF analysis, when t_0 is set, the logresponse time σ converges towards its upper bound and only decreases with movement time for the highest ratio α/β. According to the lognormal function, larger σ values lead to an earlier rise of velocity (Fig 1-B). Thus, as long as the jerk component outweighs the energy contribution to the composite function, the system is constrained to using rapid sub-systems that facilitate an initial swift increase in velocity.

For short movements (<~0.45 s), μ is independent of the ratio α/β, suggesting that that the jerk's influence on the composite function is more important than that of the kinetic energy. This observation aligns with our initial analysis of the cost functions (Fig 3-D and E). Conversely, as the movement duration increases, smaller ratios α/β result in larger and less physiological logtime delays, whereas larger ratios are accompanied by a decrease in μ. With a fixed number of activated sub-systems, an increase in movement time can only be linked to a change in the activation dynamics of the NMS, as represented by the logtime delay μ. Particularly, as movement time extends, each activation step gets slower (*i.e.*, smaller μ), thereby decreasing both the mean and median velocity of the system. For longer movements, the logtime delay μ seems more critical than σ; concurrently, the effect of the kinetic energy on the optimal control problem increases. Taken together, our findings indicate that controlling kinetic energy may be more closely associated with controlling μ, while the regulation of jerk may be linked to controlling σ.

These interpretations remain quite limited in their scope because they may depend on our optimization algorithm, the type of movement that we are studying, and our system constraints. Indeed, the values presented here are for a simple rotation, so they will most likely be quite different for more complex movements. In short, the movement type and constraints will give rise to other optimal parameter sets than the ones presented here.

3. References

Berret, B., Chiovetto, E., Nori, F., and Pozzo, T. (2011). Evidence for Composite Cost Functions in Arm Movement Planning: An Inverse Optimal Control Approach. *PLOS Comput. Biol.* 7, e1002183. doi: 10.1371/journal.pcbi.1002183

Pan, Z., Talwar, S., Plamondon, R., and Van Gemmert, A. W. A. (2019). Characteristics of bi-directional unimanual and bimanual drawing movements: The application of the Delta-Lognormal models and Sigma-Lognormal model. *Pattern Recognit. Lett.* 121, 97–103. doi: 10.1016/j.patrec.2018.05.008

Woch, A., Plamondon, R., and O'Reilly, C. (2011). Kinematic characteristics of bidirectional delta-lognormal primitives in young and older subjects. *Hum. Mov. Sci.* 30, 1–17. doi: 10.1016/j.humov.2009.10.006
